# Metapersonal Care as Philosophical Health

**DOI:** 10.1111/jep.70666

**Published:** 2026-10-12

**Authors:** Luis de Miranda

**Affiliations:** ^1^ Centre for Research Ethics and Bioethics (CRB) Uppsala University Uppsala Sweden; ^2^ Turku Institute for Advanced Studies University of Turku Turku Finland

**Keywords:** autonomy, moral psychology, person‐centred care, personhood, philosophical health, philosophy of medicine, self

## Abstract

**Rationale:**

This paper addresses the aims of the present journal concerning health philosophy and person‐centred healthcare. Person‐centred care corrected the reduction of patients to bodies and pathologies, yet its interpretation and application remain somewhat inconsistent. This article locates the inconsistency in an unresolved philosophical question: which self is the person on whom care is centred. Under pressure, a person tends to default to an inherited self rather than explore their own philosophy of life, so that an expressed preference may record the default self rather than the thinking person.

**Aims and Objectives:**

To diagnose the root of the inconsistency and to articulate metapersonal care as a necessary criterion distinguishing philosophical from naive person‐centred care.

**Methods:**

Conceptual and philosophical analysis, in dialogue with the person‐centred care and capabilities literatures, relational conceptions of autonomy, and psychological accounts of decentring, drawing on Spinoza, Murdoch, and Foucault for the theory of self‐relation.

**Results:**

What care records when it asks what the patient wants is often not the person but the epochal default self: the inherited configuration that presents itself as authentic precisely because it is immediate. Hence the paradox: the more immediately personal an expression, the more generic it may be. Between identifying with that configuration and bracketing it in the name of detachment, I develop a third position, *metapersonality*, the capacity to inhabit one's historically constituted personality without granting it sovereignty. Applied to care, this yields *metapersonal care*, helping the person to become capable of exploring the lived philosophy from which their preferences arise, and of reaffirming or revising them.

**Conclusion:**

Metapersonal care names a necessary criterion of adequate person‐centred care, one that philosophical health practices are designed to serve. Its horizon is *eudynamia*, in which cultivation of the self and composition of a shared world are no longer separable ends.

## Plain Language Summary

People's stated wishes are not always the wishes they would, on reflection, recognise as their own. We all live from habits, roles, and inherited scripts that formed long before we examined them; under pressure, illness, fear, fatigue, it is usually these that speak. Healthcare has a version of this problem. Person‐centred care rightly asks clinicians to respect patients' preferences and values, but when this is reduced to a formality, it can end up honouring only whatever a person happens to say when asked, recorded once and treated as final. This article proposes *metapersonal care* as a form of philosophical person‐centred care: care that, with the patient's permission and with no preferred outcome, supports people in examining, reaffirming, or revising the values and conceptions behind their preferences. Reaffirming an existing choice counts as fully as changing it; what matters is that the person has a thoughtful relation to what they have chosen. Philosophical health practices, which use structured sense‐making dialogue about a person's own life, are designed to support exactly this.

## 1 Introduction: The Person on Paper

Leopoldine, a hypothetical woman in her late seventies facing a treatment decision, tells the team what she has told every clinician for five years: “I will not be a burden to my children!” Her capacity is unquestioned, and the requirements of person‐centred care seem, on paper, to be satisfied. Whether they are indeed satisfied is the question of this article. It depends on a question the person‐centred tradition has largely left open: which self is the person on whom care is centred?

Leopoldine is not alone. A man drafts an advance directive at sixty, in the fear that followed his father's dying. At seventy‐four he is admitted unable to speak for himself, and the document governs. In fourteen years of appointments, nobody reopened it with him. A woman continues gruelling treatment because “I am a fighter” is on record as her personality, and no one asks whose voice the fighting is, a vow, a family role, a culture's script for illness. In a care home, a line entered years ago, “prefers to stay in her room,” quietly becomes an institution's licence to stop knocking. In many cases the requirements of a naive person‐centredness are met: a preference was elicited, recorded, and honoured. What is missed is the thinking person, and the miss is structural.

These difficulties are not idiosyncratic to a few bedsides. The critical literature has documented a persistent inconsistency in the interpretation and application of person‐centred care across settings, professions, and instruments, with continuing debate about what centredness itself requires [1, 2]. This article argues that the inconsistency and the bedside difficulties share a root, and that both call for the same remedy: a philosophical, non‐naive person‐centredness, which is named *metapersonal care*.

The argument proceeds from the bedside outward. Section 2 shows that the difficulty is general: under pressure a person tends to default to an inherited self rather than explore their own philosophy of life. It argues that what matters is whether the person can question and think what they have inherited. Section 3 returns to the clinic and shows why person‐centred care cannot be applied naively to that self. Section 4 develops the stance that answers the problem, *metapersonality*, among its philosophical and psychological neighbours. Section 5 translates the stance into metapersonal care, with its safeguards and limits, and returns to Leopoldine. Section 6 situates the proposal within the philosophical health tradition and names its horizon.

## 2 Why the Person May Not Be Speaking

Each of the cases above turns on the same difficulty, and it is not merely clinical. The difficulty at Leopoldine's bedside is a structural feature of selfhood. Everyone lives from assumptions that are most often implicit: a set of orientations toward the body, others, work, mortality, and what is worth wanting, acquired long before it is examined and rarely revisited. Philosophical health implies that the measure of a philosophy of life is what remains of it when the person becomes too personal, that is, under irritation, fatigue, pain, illness, fear, pampering or the pressure of a decision that cannot be postponed. A philosophy held as doctrine, whether learned in a seminar or absorbed from a family, may be perfectly articulable without governing critical moments. Under pressure the articulable version tends to give way to a generic script, and what speaks is defensiveness, comparison, inherited behaviour, or a sentence rehearsed for decades.

This journal has been built on the conviction that gaps between theory and practice are not embarrassments to be hidden but the very object of evaluation. The possible gap between a person's articulated philosophy and their operative mode of existence demands a diagnosis rather than a shrug, and it deserves one at the bedside no less than in the seminar room. What bears the weight of a decision, when the articulated philosophy does not, is the epochal default self, and I will argue that its unexamined predominance is the root of the inconsistency of person‐centred care. The path from that diagnosis back to the clinic runs through a concept I propose to call metapersonality, and its clinical translation, metapersonal care. My claim, condensed in the title, is that metapersonal care names a necessary criterion of adequate person‐centred care, one that philosophical health is designed to serve.

What does appear when the sophisticated person “becomes personal”? The modern sensibility, formed by the romantic equation of authenticity with proximity to feeling, tends to assume that what surfaces under pressure is the real self, the person beneath the persona. The assumption has a history: since Rousseau, the inner voice has been credited with a truthfulness that convention and reflection allegedly corrupt, so that immediacy itself became the mark of the genuine. I want to argue nearly the opposite. What presents itself as confession about “simply how I am and feel” can reveal the most socially manufactured layer of a person: an assemblage of therapeutic individualism, romantic scripts of self‐expression, competitive status consciousness, familial roles rehearsed since childhood, and, increasingly, preferences reinforced by algorithms whose business model is the profitable stabilisation of the average self. The supposedly irreducible “me” is highly reproducible. Hence a paradox that reverses the modern equation: the more spontaneously personal I become, the more generic I may become.

Each of these forces deserves a moment, because their convergence is what makes our default self historically distinctive. Therapeutic individualism, whose ascendancy Rieff diagnosed more than half a century ago, often teaches that the inner life is the final authority and that expressing it is psychological health [3]. The culture of authenticity carries, as Taylor has shown, a genuine moral ideal, being true to oneself; but the ideal degrades when the self is treated as something already there, waiting to be consulted, rather than as something a person works out philosophically. Consulted in that spontaneous spirit, the self often returns what has been put into it, and being true to oneself becomes a way of ratifying one's inheritance or conditioning under the name of freedom [4]. Today algorithmic personalisation closes the loop: artificial dialogic systems optimised for engagement learn the existing configuration and feed it back, so that the default self is no longer merely inherited from family and epoch but actively curated by cognitive averaging infrastructures with a commercial stake in its vulnerability. The result might be called a false generic authenticity: a personalism of the lowest common denominator, experienced from within as singular expression.

The paradox should not be misread as a claim of uniformity. There is a level of singularity in accumulated personal experience, and default selves can differ sometimes strikingly: temperament, biography, and milieu distribute the common materials differently, and what ordinary language calls someone's character, the recognisable way a person is when no one, including themselves, is steering, is precisely this individuated sediment. What the paradox targets is not the existence of individual character but the inference from distinctiveness to authenticity: a singular opinion, held without thoughtful articulation, is often no more the person's own view than a common available one; distinctiveness is not evidence of authorship.

The self is not an inner theatre furnished by outside influences. Read through the lens of embodied, embedded, enactive, and extended (4E) accounts of cognition, the default self is itself a distributed achievement: sedimented partly in external scaffolds, familial routines, institutional files, or recommendation systems, which is precisely why it is so reproducible [5]. The person's mind is embodied as well as embedded. The gut feeling of authenticity, the somatic conviction that this reaction is truly mine, is sometimes the body's recognition of a habit rather than a verdict about the self. Metapersonality, correspondingly, will not be an inner representational state but an enactive, relational performance, a reflective way of engaging one's distributed constitution.

The intuition that the spontaneous self is sedimented rather than deeply personal has a distinguished lineage. Bergson contrasted the *moi superficiel*, the socially sedimented self of ready‐made states and public language, with a deeper stance that ordinary life almost never solicits [6]. Heidegger described the dictatorship of *das Man*, the anonymous “one” that supplies our reactions [7]. Bourdieu's habitus named the incorporated social and familial history that generates everyday preferences beneath the level of deliberation [8]. Freud's account of everyday automatisms and, in contemporary psychology, dual‐process descriptions of automatic and reflective processing describe a similar phenomenon from other sides [9, 10]. The impersonality of the so‐called personal construct is well attested and it should long since have reached the settings where preferences are elicited and recorded as final. But it has not.

One clarification is essential before proceeding: the difference that matters is between constitution and fixation: between an inheritance or habit a person can question or transform, and one that has become opaque to them, insulated from reasons and changed circumstances. The aim of metapersonal care is not the recovery of a pure chooser but the widening of a philosophical relation with life, beyond both personality and impersonality, as we will see below.

## 3 The Trap in the Clinic

Person‐centred care arose as a necessary corrective. Against a biomedicine that addressed organ systems and survival metrics, the person‐centred movement insisted that the one who is ill is a person: a bearer of narratives, values, preferences, and a biographical world that treatment either sustains or violates [11]. The journal that publishes this article has been one of the principal sites of that insistence. The question I wish to raise is internal to the movement's partial success. It concerns not whether care should centre on the person but which person it thereby centres on.

Notable philosophical work in the tradition has already seen that the concept needs deepening. Mitchell and Loughlin distinguish person‐centredness as a desirable ethical add‐on to good biomedical practice from person‐centredness as a fundamental conceptual shift, and they argue for the latter: personal life understood as an ongoing, inter‐creative interaction with other people, meanings, values, and environments [11]. The capabilities strand also gestures toward the present proposal. Entwistle and Watt argue that treating patients as persons means recognising and cultivating their capabilities, including capabilities for autonomy, on an account that explicitly considers people with limited decision‐making capacity [12]. The present argument supplies a diagnosis of what the capability for self‐relation is up against.

The knot of inconsistency with which this article began can now be diagnosed rather than merely observed. Some conclude that person‐centred care is a fallacious notion altogether, others that its vagueness is manageable or even productive [1, 2]. The critique that follows is aimed at what may be called the thin implementation of person‐centred care: the administratively reductive form, familiar in practice, in which centring on the person becomes recording and honouring the preferences of the unexamined self. Personalism assumes that the person on whom care is centred is a given authentic self, available to be consulted, whose expressed preferences constitute the voice that paternalism had silenced. Elsewhere I have argued that institutional bioethics tends to treat life as a generic given whose clinical preservation exhausts ethical attention, leaving the *bios ethikos* question of which form of life we are dealing with unasked [13]. A structurally analogous displacement operates here: care centres on the person as given, and the question of which self has been found does not arise.

The point must be stated fairly, because clinical practice is not naive about values. Explicit values‐clarification methods are integral to some patient decision aids [14], and serious‐illness conversation guides ask about goals, fears, sources of strength, and what matters most [15]. These practices support reflection, but they tend to remain, by design, decision‐specific and option‐oriented: they clarify the values relevant to a choice among presented options, and rarely address the person's broader relation to their practical identity, the inherited narratives, the philosophical worldview, and the horizon of possibility within which those values became theirs. If the spontaneous self is substantially an epochal assemblage, then what is retrieved at the bedside will often be the sedimented voice of inherited scripts, familial roles, reactive ressentiment, cultural narratives of fighting on or of not being a burden. Respect for autonomy, at this limit, becomes respect for sedimentation. The recording apparatus may remain faithful here to the most fixated and superficial layer of the patient. And the more sincerely immediacy is honoured as authenticity, the more reliably the generic might be ratified. The paradox of Section 2 returns in institutional form: the more person‐centred the procedure, in this preference‐recording sense, the less of the person it may serve.

Relational conceptions of autonomy have argued that the liberal picture of the self‐transparent chooser is a fiction, that selves are constituted in and by their relations, and that oppressive socialisation can compromise autonomy from within [16]. The argument of this article is a redirection of that insight. The constitution in question is not only oppressive socialisation but the ordinary epochal sedimentation of every self; there is no unmarked chooser to whom the relational critique does not apply. Some relational accounts, notably those centred on oppressive socialisation, ask whether a preference is truly the person's, given its social pedigree; metapersonal care asks whether they have a philosophically healthy distance to it, regardless of social pedigree. The practical conclusion is philosophical care hosting the dialogue in which the person gains a thoughtful and compossible relation to their preferences, as we will see below.

This is not a licence for paternalism. The paternalist claims to know the patient's good better than the patient. The present argument observes that the patient's thinking may transcend the patient's default personal configuration, and that a care which freezes the person at this default configuration respects too little, not too much. If anything, it is the preference‐recording model that harbours a quiet paternalism, since it decides on the patient's behalf that they are identical with their self‐sediment. The disagreement with the naive preference‐recording model is therefore not about the authority of the person, but whether the person is coextensive with the self that presents or includes the capacity to examine and exceed that self. Person‐centred care answered the first question correctly against biomedicine: the person is more than the body. But we must add that the person is also more than the personality.

## 4 Impersonal, Personal, Metapersonal

What then should the person do? The most venerable, and dangerous, response to the prevalence of the default self is subtraction, stoic‐like refuge into the impersonal. What I call the impersonal stance is an ideal type, not a scholarly account of Stoicism, because the ideal type is culturally pervasive, especially in contexts of institutional care. It hopes to gain distance from the so‐called passions through rational reappraisal: the event is not in my control, my judgement about it is. The strategy has merits, but as a response to the default self it remains structurally reactive.

What is bracketed in the popular stoic attitude continues to organise the field from which it has been excluded; here the discipline of impersonality is defined, at every moment, by the personal material it holds at bay. Nietzsche pressed the point without mercy: the ideal of apatheia is itself a passion in disguise, an ascetic will turned against the affects, and its disciplined serenity can become avoidance or even revenge on life [17]. A detachment that is reactive remains bound to what it renounces, and what is repressed with discipline tends to return without it: in irritation, in irony, in the passive aggression of the conspicuously calm. The forced impersonal stance can moreover become a further persona, and it often does: the composure that is itself a performance, more or less abandoned in private.

The opposite response is identification. On this view, the reactions or narratives of the spontaneous and emotional self are to be honoured as authentic, and the task is to express or follow them more fully. Section 2 has given reasons to doubt this. If the spontaneous configuration of the self is substantially a superficial product, then fidelity to it is not self‐possession but a familiar form of dispossession.

The stance I propose is neither loosely spontaneous nor forcibly stoic: metapersonality is the capacity to thoughtfully and purposefully inhabit one's socially and historically constituted personality, without granting it sovereignty. The metapersonal subject does not suppress the personal and does not obey its default mode. They may remain warm, humorous, desirous, ambitious, embodied, singular by experience; what changes is that the immediate configuration no longer dictates the direction of responsibility. One can notice that recognition is being sought, that a slight has registered, that the wish to win has arisen, without either moralising these movements or executing them.

DeCicco and Stroink introduced and psychometrically studied the metapersonal self‐construal: a sense of identity extending beyond the individual and the relational toward humanity, life, or a larger whole [18]. Spinoza's transition from passive affects to adequate activity describes the process: an affect ceases to be a passion once its causes are understood *sub specie aeternitatis* [19], under the aspect of a larger network that involves not only the person, but the earth, webs of work, and society. Murdoch's unselfing names the moral core, the patient displacement of the “fat relentless ego” through attention to what is not oneself [20]. Foucault's work on the care of the self supplies the practical frame, the self as material for a deliberate art of lived composition rather than a reactive datum to be expressed [21].

Metapersonality need not suggest a superior observing ego, a second personality installed above the first: it is less the possession of a higher identity than the widening of identification as such. There is no distinct second self who supervises a separated first; there is a first self held more philosophically. A supervisory ego has contents, positions, and prestige to defend; the confident claim to have achieved metapersonality could itself be a datum for suspicion, since it has the grammar of a possession, and possessions are what the default self collects.

Metapersonality is less a higher storey in the hierarchy of attitudes than a changed relation to the whole structure. The hierarchical account of the will holds that a person is constituted by their second‐order volitions: I am the desires I endorse desiring [22]. The account captures something real, but the hierarchy does not by itself escape the epoch, for second‐order attitudes are as available for sedimentation as first‐order ones; the culture that scripts my desires also scripts my endorsements, and a wholehearted identification with an epochal configuration can still be an epochal fact.

Metacognition, the monitoring of one's own cognitive processes [23], is also relevant but not sufficient: the skilled rationaliser can be a virtuoso of metacognition in the service of the default self. Closest constructs to what is meant in this article come from third‐wave cognitive and acceptance‐based therapies: decentring, cognitive defusion, and self‐as‐context, a family critically reviewed as involving a shift to an observing relation in which thoughts and feelings are had rather than obeyed [24]. Self‐as‐context in particular, the “I” as perspective rather than content, suggests the capacity is trainable. What the present proposal adds is a normative concern with authorship and possibility rather than with symptom reduction or psychological flexibility alone; and, in the clinical development below, a dialogical practice in which the carer's interpretive position is itself subject to examination.

The criterion of Section 2 can now be restated with more precision. Metapersonality must be a regular process and is never achieved once and for all. As Hadot showed, the ancients took the decisive examination to occur in the texture of a lived day [25]. What a held philosophy requires in order to reach conduct is a cultivated relation to one's own constitution and compositions, and such a relation is acquired in philosophical dialogue or reflection, rather than in doctrine, which is why the practices discussed in Section 6 are conversations and not curricula. The next section translates this stance into care.

## 5 Metapersonal Care

Metapersonal care is care addressed to the person as capable of exceeding their constituted personality, where exceeding means the philosophical freedom to examine, contest, transform, or reaffirm under the expanded aspect of personal embodiment, earthly experience, enactive engagement, and societal belonging. So understood, metapersonal care is not a successor to person‐centred care but a necessary criterion of it: the test that distinguishes adequate person‐centred care from its thin implementations. It includes everything the tradition rightly demanded, the narrative, the values, the biographical world, and it adds the refusal to reduce the person to their current unexamined configuration.

In administratively thin implementations of shared decision‐making, elicitation may be reduced to recording preferences and attempting to honour them. Metapersonal care would not abolish this procedure but change its temporality and its address. Elicitation asks what the person wants; metapersonal care creates the conditions under which the person can reopen a capability for eudynamia, a good relationship with the possible across domains that are compossible to the person: earth, work, and society [13].

Narrative medicine has shown that restoring narrative coherence can be therapeutic without being curative [26]. Metapersonal care generalises the point beyond narrative: what illness disrupts, and what preference‐recording cannot reach, is the person's purposeful relation to their own possibilities, the sense that existence may still unfold into meaningful new forms or directions [27]. A consent conversation conducted metapersonally does not press the patient toward any particular choice; it gives the choice time and dialogue enough that the embodied, extended, enactive and embedded person, and not only their reactive sediment, can make it. The scripts of fighting on, of not being a burden, of what a mother or a man or a modern stoic does, are held in view with the patient until they can set them at a healthy distance.

Remember Leopoldine and her stated position of not being “a burden”. A metapersonal conversation does not contest her statement. It gives it air. Asked, with her permission, whether she would like to say more about the sentence that has travelled with her for five years, she speaks about her own mother's difficult dying, six decades earlier, and the vow of a teenager never to weigh on anyone. She may, after discussing her bodily sense, sense of self, sense of belonging, sense of the possible, sense of purpose, and her worldview, reaffirm exactly the choice she began with, and if she does, the conversation has succeeded as fully as if she had revised it. What has changed is not the content of the decision but the lucidity of its holder: the vow is no longer only holding her; she is also holding the vow.

Chronic illness and old age make the stakes institutional. A person living for decades with a progressing condition is asked, repeatedly, to state preferences that institutions then treat as their settled identity, which is easier to administer. In elder care the reduction is often complete: the resident becomes their documented preferences, their file the frozen portrait that efficiency prefers to the living original. Metapersonal care in these settings treats the eighty‐year‐old as still capable of surprising their personal file. Much of this asks first for a different quality of attention, and then for the institutional conditions that make such attention possible: protected time, continuity, and training in deep listening and sense‐making dialogue.

A qualification must now be made explicit. While metapersonality names a capacity, metapersonal care names a clinical stance that remains binding regardless of the person's present reflective capacities. The capacity may be partial, fluctuating, and relationally supported rather than individually exercised; a person with dementia, delirium, severe aphasia, or intellectual disability is not less a person on this account, and nothing here makes reflective distance a condition of being owed the best possible care. Where reflective revision is unavailable, metapersonal care does not lapse; it may persist as the refusal to reduce anyone to a file, a diagnosis, a recorded preference, or a cognitive deficit, and as the relational work, with families, carers, and advocates, of keeping a person's compossibilities in view when the person cannot decide alone. At this point the proposal joins supported decision‐making and rejoins the capabilities account, which was framed with precisely such persons in mind [12].

More generally, metapersonal care cannot be reduced to checklist compliance, a mandatory second question, or a documented “exploration of value provenance” appended to the consent form. A proceduralised loosening is a contradiction in terms: the checklist would be administered by and to default selves. What can be cultivated is a quality of address, teachable the way clinical judgement is teachable, through practice, supervision, and sense‐making dialogue rather than rigid protocol. This belongs in the formation of the carer. What success looks like must also be stated, since by design it need not appear in the choice itself. When reaffirmation is the outcome, what has changed is the person's relation to the choice and to what remains possible: the decision now composes with the life it belongs to and with the others it implicates. This is not the adaptive coherence of salutogenesis, a better fit between person and circumstance, but a eudynamic one, a widened and philosophically examined relation to the possible and the compossible across person, earth, work and society (PEWS) [13].

Now, metapersonal care is complete only with safeguards, as exploration can itself exert pressure. Five safeguards are constitutive of the practice rather than optional refinements. *Permission*: the history of a value is opened only with explicit consent, and the patient retains a plain right to decline philosophical exploration altogether. *Outcome neutrality*: reaffirmation and revision are honoured equally, and a preference is never examined merely because the clinician dislikes where it leads. *Asymmetry acknowledged*: the encounter is structured by unequal epistemic power, and the practice must be designed with that structure in mind. *Reciprocity*: the carer's professional persona, with its scripts of competence, efficiency, and emotional management, may be as epochal a formation as any patient's personality, and philosophical supervision should exist to examine it, including the carer's worldview and preferred outcomes. And *restraint*: the carer must not become the superior observer who sees through the patient's scripts. Clinical relationships are never symmetrical; what metapersonal care requires is reciprocal reflexivity.

## 6 The Horizon of Eudynamia

A capacity that must be held daily requires practices. The philosophical health approach, as it has developed in this journal and beyond, can be read as a working tradition of such practices: structured philosophical dialogues designed not to record the preferences of the default self but to accompany the person through six elements of their existence, bodily sense, sense of self, sense of belonging, sense of the possible, sense of purpose, and philosophical sense, until the configuration they arrived with is no longer a reactive position; the SMILE_PH method (Sense‐Making Interviews Looking at Elements of Philosophical Health), detailed in this journal, is one existing operationalisation of such dialogue [28, 29]. Qualitative interviews with persons living well after spinal cord injury identified processes of reflective self‐reinvention and transformed relations to possibility; these preliminary findings support the relevance of the framework proposed here, but they do not fully establish that philosophical‐health dialogue causes such changes, which remains an open question for intervention research [30]. Described from the outside, these are person‐centred methods. Described more precisely, they are metapersonal in design: their object is not the person as found but the person as capable of a transindividual stance.

The horizon of such care has been called *eudynamia*: the stabilised good relation to the possible and the compossible (what is possible together), as the orientational concept of an approach to bioethics concerned with meaningful forms of life rather than survival alone [13, 31]. Metapersonality is the personal condition of eudynamia according to the formula T = PEWS + C. In that formula, ethical thinking (T) emerges from an integrative reflection on the four lived domains of Person, Earth, Work, and Society (PEWS) plus C, the creative openness that exceeds any settled systematisation [13]. The self‐relation described in this article is not only philosophical but also augmentative; its natural extension is the augmentation of a shared world, since a person no longer defending a default configuration has attention to spare for what can be composed with others, or is considered by others as part of their eudynamic becoming. Cultivation of the self and care for the common world, sometimes opposed in the ethics of authenticity, cease at this horizon to be separable ends.

## 7 Conclusion

Person‐centred care fails if it grants sovereignty to the epochal default self. The remedy proposed here is a single capacity with two faces. As metapersonality, it is the self‐transcendent ability to inhabit one's constituted personality without being commanded by it, and with a larger existential belonging in view. As metapersonal care, it is the criterion of philosophically healthy person‐centred care: the address that treats the patient, the families, the carers, as capable of examining, reaffirming, or revising the self from which their preferences arise, under the aspect of our personal, earthly, professional and societal distribution. The criterion with which this article began can accordingly be given its clinical form: the measure of a practice of care is what of the person remains addressable when the default self has finished speaking.

Three lines of work follow. *Conceptually*, the boundaries drawn above between metapersonality and its neighbours, hierarchical volition, relational autonomy, self‐as‐context, deserve fuller treatment than this article could give them. *Empirically*, the claims advanced here are still partly hypothetical: that sense‐making dialogical practices in general, and the SMILE_PH method in particular, produce a lucid widening rather than a mere preference change, should be testable, and newly developed instruments for assessing existential dimensions of health, whose validation is itself ongoing, offer one starting point [32]. *Pedagogically*, if the quality of address is teachable through formation rather than protocol, curricula for clinicians and counsellors should be designed or implemented, and evaluated accordingly.

What remains is the work of showing in detail, setting by setting, what care gains when it realises it is not only embodied in individuals, but extended across earthly protocols, both natural and technological, enacted in work practices, embedded in societies, and constantly reexamined and reinvented.

## Conflicts of Interest

The author is guest co‐editor of the special issue in which this article appears and was fully recused from its particular editorial handling. The article was assessed by two independent reviewers.

## Data Availability

Data sharing not applicable to this article as no datasets were generated or analysed during the current study.
